# The complete mitochondrial genome of a polyphagous insect: *Colasposoma dauricum* (Coleoptera: Chrysomelidae: Eumolpinae)

**DOI:** 10.1080/23802359.2021.2010614

**Published:** 2021-12-23

**Authors:** Mi Shen, Yue Fu, Jiaojun Yu, Jun Fu, Yunli Xiao

**Affiliations:** a College of Biology and Agricultural Resources, Huanggang Normal University, Huanggang, China; b Hubei Zhongke Research Institute of Industrial Technology, Huanggang, China; c Hubei Key Laboratory of Economic Forest Germplasm Improvement and Resources Comprehensive Utilization, Huanggang, China

**Keywords:** Leef beetles, *Colasposoma dauricum*, mitochondrial genome, phylogeny

## Abstract

*Colasposoma dauricum* Mannerheim, 1849, is an important insect pest distributed in most areas of China. The complete mitochondrial genome of *C. dauricum* was sequenced and analyzed. The phylogenetic relationships between *C. dauricum* and other 10 species in the superfamily Chrysomeloidea were reconstructed using maximum likelihood (ML) methods based on the concatenated nucleotide sequences, the phylogenetic analysis showed that *C. dauricum* is closely related to *Basilepta fulvipes* in the same subfamily.

*Colasposoma dauricum* (Coleoptera: Chrysomelidae: Eumolpinae) is a polyphagous insect widely distributed in North and Northeast China (Montagna et al. 2016). In China, the native area, it feeds on different species of Convolvulaceae, Asclepiadaceae and Apocynaceae (Ahn and Lim 1991; Jolivet and Hawkeswood 1995). Pest survey in *Artemisia argyi* plants, reported serious damage caused by *C. dauricum*. Previously published mitochondrion genome of *C. dauricum* GenBank: KY039104.1 was partial. In this study, the mitogenome was complete, supplementing the sequences of NADH1, tRNA-Leu, 16S RNA, tRNA-Val and 12S RNA.

The specimen in this study was collected from Huanggang City (Hubei Province, China) (115°45′78″E, 31°13′94″N) in 2020 and deposited in the Biodiversity Herbarium of Huanggang

Normal University (no. HGNU-QA1; Email contacts of the people in charge of the collection: shenmi@hgnu.edu.cn). The adult sample, with total weight of about 0.2 g, was used for experiments. Salting-out method was used to extract the sample DNA. The obtained pure DNA was used to construct a genomic library and to start high-throughput sequencing using Illumina HiSeq X. The sequences were assembled using SPAdes v3.11.19 (http://cab.spbu.ru/software/spades/) and annotated using MITOS web server.

PhyloSuite (Zhang et al. 2020) was used for the phylogenetic analyses with several plug-in programs: MAFFT (Katoh and Standley 2013) using ‘-auto’ strategy and codon alignment mode. PartitionFinder2 (Lanfear et al. 2016) was used to select best-fit partitioning schemes and models using AICc criterion. The number of bootstrap replicates was 5000, and Maximum likelihood phylogenies were inferred using IQ-TREE (Minh et al. 2013; Nguyen et al. 2015). The topology of the trees were visualized and edited in iTOL (Letunic and Bork 2019).

The complete mitogenome of *Colasposoma daurium* is 15,490 bp in size (GenBank accession number: MW354515). It includes 13 protein-coding genes (PCGs), 22 tRNA genes and 2 rRNA genes, a total of 37 genes. There are 14 gene overlapping regions in the genome. The total overlapping length is 459 bp, ranging from 1 to 345 bp. The longest overlapping region (345 bp) located between *16 s rRNA* and *trnV*. There are 6 intergenic spacers with a total length of 44 bp, the longest intergenic 22 bp located between *trnS* and *ND1*. The genomic nucleotide composition is A:T:C:G = 40.11%:34.14%:14.90%:9.76%. The total length of 13 PCGs in the mitochondrial genome is 11,096 bp. The start codons of twelve PCGs were the same with that of *C. dauricum* GenBank: KY039104.1. The newly announced gene, *nd*1, began with TTA. The stop codons of nine genes (*nd2, cox1, cox2, atp6, nd3, nd5, nd4L, nd6, and cytb*) are the same as those previously published. The stop codon of *atp8*, *cox3* and *nd4*, changed from ATA to TAA, GGT to ATA, CAT to TAT, respectively. The gene, *nd1*, is terminated by CAA. The length of tRNA genes ranged from 63 to 72 bp, 1459 bp in total length. The length of 12S rRNA and 16S rRNA are 833 and 1660 bp in length, respectively.

The concatenated nucleotide sequences of 13 PCGs from nine leaf beetles in family Chrysomelidae and two outgroups (*Anoplophora glabripennis* NC008221 and *Batocera lineolata* NC022671 in family Cerambycidae) were used to construct the phylogenetic tree. The phylogenetic analysis showed that *C. dauricum* is closely related with *Basilepta fulvipes* MT627597, both belong to the same subfamily Eumolpinae, which is in accordance with the traditional morphological classification (Figure 1). This result is also consistent with previous phylogenetic analyses in other Eumolpinae insects (Feng et al. 2020).

**Figure 1.**
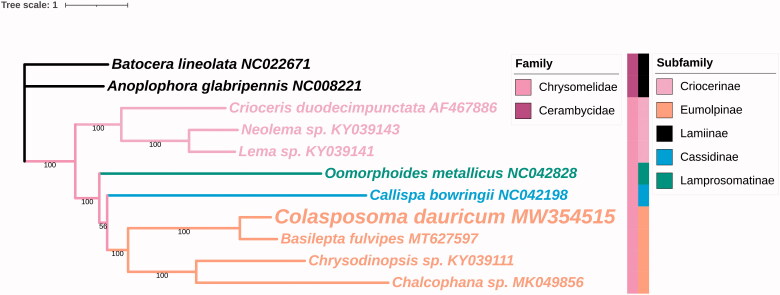
Phylogenetic relationship of 11 species in Chrysomeloidea based on the concatenated data set of 13 protein-coding genes. NC008221 and NC022671 were shown as outgroups. Number under each node indicates the ML bootstrap support values.

## Data Availability

The data that newly obtained at this study are available in the NCBI under accession number of MW354515 (https://www.ncbi.nlm.nih.gov/nuccore/MW354515). The associated BioProject, SRA, and Bio-Sample numbers are PRJNA758734, SRP334637, and SAMN21033541 respectively.
